# Photoluminescent copper(I) iodide alkylpyridine thin films as sensors for volatile halogenated compounds

**DOI:** 10.3389/fchem.2023.1330227

**Published:** 2023-12-11

**Authors:** Mahboubeh Jamshidi, Joey Bouheriche, James M. Gardner

**Affiliations:** Department of Chemistry, Division of Applied Physical Chemistry, KTH Royal Institute of Technology, Stockholm, Sweden

**Keywords:** copper(I) iodide, N-donor ligand, thin film, sensor, time-dependent photoluminescence

## Abstract

The paper presents the fabrication and characterization of [CuI(L)]_n_ thin films, where L represents various alkylpyridine ligands including 4-methylpyridine, 3-methylpyridine, 2-methylpyridine, 4-^t^butylpyridine, 3,4-dimethylpyridine, and 3,5-dimethylpyridine. The thin films were synthesized by exposing the corresponding ligands to CuI thin films through vapor deposition. The coordination reactions occurring on the films were investigated using PXRD and time-dependent photoluminescence spectroscopy, and a comparison was made between the structures of the thin films and the corresponding powder phases. The films showed primarly blue emission (λ_em_ = 457–515 nm) and polymeric structures with excited state lifetimes ranging from 0.6 to 5.5 μs. Significantly, the studied compounds exhibited fast reversible luminescence quenching when exposed to vapors of dichloromethane and dibromomethane (15 and 30 min respectively), and the luminescence was restored upon re-exposure to the alkylpyridine ligand (after 20 min). These findings indicate that these thin films hold promise for applications as sensors (with sensitive and reversible detection capability) for volatile halogen-based compounds (VHC).

## 1 Introduction

Copper iodide (CuI) has been a topic of interest for material scientists due to its unique optoelectronic properties and applications (Yu et al., 2017; Chen et al., 2018; Busch et al., 2019; Ki et al., 2021; Schlachter et al., 2021). CuI is a wide bandgap semiconductor (3.1 eV) which makes it an attractive component of optoelectronics, such as solar cells, photodetectors, and light-emitting diodes (LEDs) (RUSOP et al., 2004; Sankapal et al., 2004; Wang et al., 2017; Yin et al., 2021; Vora-ud et al., 2022). As well, CuI readily reacts with volatile organic compounds and may act as a reversible sensor for the detection of gases and monitoring temperature and pressure (Safko et al., 2012; Kondo et al., 2020; Dai et al., 2023; Jia et al., 2023).

Copper(I)-halide complexes exhibit structural diversity that is coupled to their photoluminescence (Boden et al., 2021; López et al., 2022; Murillo et al., 2022). Simple combinations of copper iodide and pyridine-based ligands in varying ratios yields compounds ranging from mononuclear [CuI(3-Methylpyridine)_3_] to blue emissive stair-step polymer [CuIpy]_∞_. The coordination sphere is largely determined by electronic and steric effects as well as crystallization solvent and concentration in solution. For instance, solutions of *{CuIpy}*_*n*_ (where *py* = pyridine or pyridine derivatives) primarily exist as tetrahedral clusters, *[CuIpy]*_*4*_ unless it is in the presence of high concentrations of *py* leading to the formation of mononuclear or multinuclear structures. Conversely, crystalline solids isolated from such solutions may take the form of either tetranuclear or polymeric structures, depending mainly on the crystallization conditions, particularly solvent (Ford et al., 1999; Cariati et al., 2000; Näther and Jeß, 2002; Zhang et al., 2014).

Among the copper(I)-halide complexes, the compounds containing N-heteroaromatic ligands have garnered attention due to their versatility and impressive luminescent, photophysical, and electrochemical properties (Liu et al., 2015; Kirakci et al., 2017; Kobayashi et al., 2020; Egly et al., 2021). Their unique features render them optimal candidates and establishing them as a compelling research area for scientists. The cost-effectiveness and bright emissivity of copper(I)-halide complexes in the solid-state makes them a preferred choice. By judicious selection of the ligands, the emission wavelength of the complexes can be fine-tuned from 390–650 nm, underscoring their potential in sensing and identifying N-heteroaromatic compounds (Ohara et al., 2014; Ohara et al., 2017; Chen et al., 2023). Copper(I)-halide complexes are well known for their vapochromism, which could be applied for sensing molecules in the gas phase. Copper(I) materials undergo changes in their photoluminescence upon exposure to volatile compounds, which reversibly induce structural changes. The reversible structural changes and luminescent behavior make them candidates for sensor applications (Chai et al., 2015; Grupe et al., 2020; Tang et al., 2023).

The effectiveness of complexes in sensors relies heavily on producing thin films (Klochko et al., 2019). By reducing the density of the complex molecule through vapor exposure, changes in the complex can be more easily observed. Conversely, powdery samples require prolonged vapor exposure spanning several hours to exhibit changes in luminescence color (Touzani et al., 2011; Lee et al., 2022). This inconsistency is attributed to the difficult diffusion of vapor within densely packed crystal lattices.

The primary objective of this study is to explore the response of CuI films to various pyridine derivatives. To track any changes, we will employ time-dependent photoluminescence spectroscopy. Subsequently, we assess the responsivity of thin films to CH_2_Cl_2_ and CH_2_Br_2_ vapors to assess their effectiveness as sensors for halogenated organic molecules.

## 2 Experimental

### 2.1 Materials

CuI (99%), CuSO_4_·5H_2_O, (98%), Na_2_S_2_O_3_ (99.99%), KI (99%), 4-methylpyridine (99%), 3-methylpyridine (99%), 2-methylpyridine (98%), 4-tbutylpyridine (98%), 3,4-dimethylpyridine (98%), 3,5-dimethylpyridine (98%), dibromomethane (99%), acetonitrile (99.8%), and CDCl_3_ (99.8%) were purchased from Sigma Aldrich. Anhydrous dichloromethane (99.8%) and microscope slides were purchased from VWR Sweden. All chemicals were used as received without any further purification.

### 2.2 Fabrication of thin film

CuI was first deposited onto glass substrate by spin-coating. The solution for spin-coating was prepared by dissolving 0.10 g (0.525 mmol) of CuI into 10 mL of acetonitrile. Spinning condition was 3,000 rpm for 20 s. Subsequently, the thin film was heated using a hot plate set at 61°C to evaporate the solvents. Spin-coating was repeated 4 times for the same film to grow the thickness of the layer.

Another method for fabricating CuI thin films is the SILAR (successive ionic layer adsorption and reaction) method, in which a glass substrate is immersed separated cation and anion aqueous solutions with a water wash between steps, as described in the literature (Dhere et al., 2010). For the cation solution, 1.05 g (4.2 mmol) of CuSO_4_ 5H_2_O was dissolved in 42 mL of water in a 50 mL beaker. Also, 94.8 mg (0.6 mmol) of Na_2_S_2_O_3_ was dissolved separately in 6 mL of water before mixing with the copper solution. The cation precursor solution was clear blue in color. The anion precursor, on the other hand, was a transparent KI solution in a 50 mL beaker that contained 199.2 mg (1.2 mmol) of KI dissolved in 48 mL water. Two beakers containing distilled water were placed between the cation and anion precursor for washing.

One SILAR cycle was completed by immersing the substrate into the cation precursor for 5 s and into the anion for 20 s. The washing steps each lasted for 3 s and removed excess ions from the substrate. The cycles were repeated 30 times to obtain a favorable film thickness.

Next, the CuI film was placed in an empty 2.0 mL vial, which was further placed in a 10.0 mL vial containing the ligand solutions. The 10.0 mL vials were sealed, which allowed the ligand solution in the container to vaporize and react with the CuI thin film and form the [CuI(L)]_n_ compounds as thin films. The [CuI(L)]_n_ thin films were emissive and further characterized by PXRD.

To test the vapochromism of the films in the presence of halogenated organics, thin films of copper(I) iodide complexes on glass slides were suspended in a quartz cell and a few drops of the desired CH_2_Cl_2_ or CH_2_Br_2_ solution was added inside the cell without any direct contact between the film and the solution.

### 2.3 Characterization

X-ray diffraction (XRD) patterns were measured on X'Pert PRO, PANalytical using CuKα radiation (λ = 1.5406 Å) to analyze the purity of the prepared sample and to determine the phase and crystal structure of the prepared materials. ^1^H-NMR spectroscopic experiments were performed with a Bruker DMX-500 MHz spectrometer using CDCl_3_ as reference solvent.

### 2.4 Photoluminescence measurements

Photoluminescence measurements were carried out on a Fluorolog FL 3-22 spectrometer (Horiba Jobin Yvon, Longjumeau, France), equipped with a double excitation monochromator, a single emission monochromator (HR320) and a R928P PMT detector. A continuous xenon lamp (450 W) was used for steady state measurements. A Delta Diode (λ_ex_ 360 nm) was employed as the pulsed source for TCSPC lifetime acquisition. Luma 40 heat exchanger connected to TC1 temperature controller was used for temperature adjustment.

## 3 Results and discussion

### 3.1 Synthesis and characterization of thin films

**FIGURE 1:**
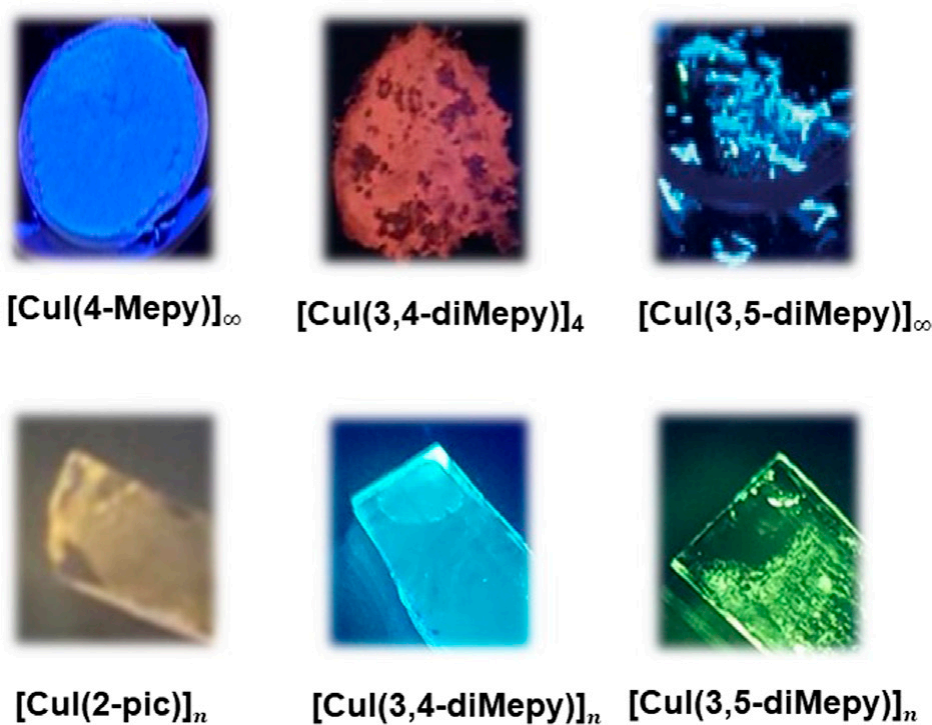
Photos of CuI complexes (top) in powder form and (bottom) on film under irradiation at 365 nm.

Synthesis of powder samples of [CuI(L)]_n_ (*n* = 4 or ∞) where L = 4-methylpyridine (4-Mepy), 3-methylpyridine (3-Mepy), 2-methylpyridine (2-Mepy), 4-^*t*^butylpyridine (4-^*t*^Bupy), 3,4-dimethylpyridine (3,4-diMepy), and 3,5-dimethylpyridine (3,5-diMepy) were reported previously with their crystal structures (Die Strukturchemie der KupferI et al., 1980; Rath et al., 1986; Cariati et al., 2000; Cariati et al., 2005; Parmeggiani and Sacchetti, 2012; Kitada and Ishida, 2014). They exhibited strong blue emission to blue-green, yellow, or red according to their structures as shown in Figure 1.

**FIGURE 2:**
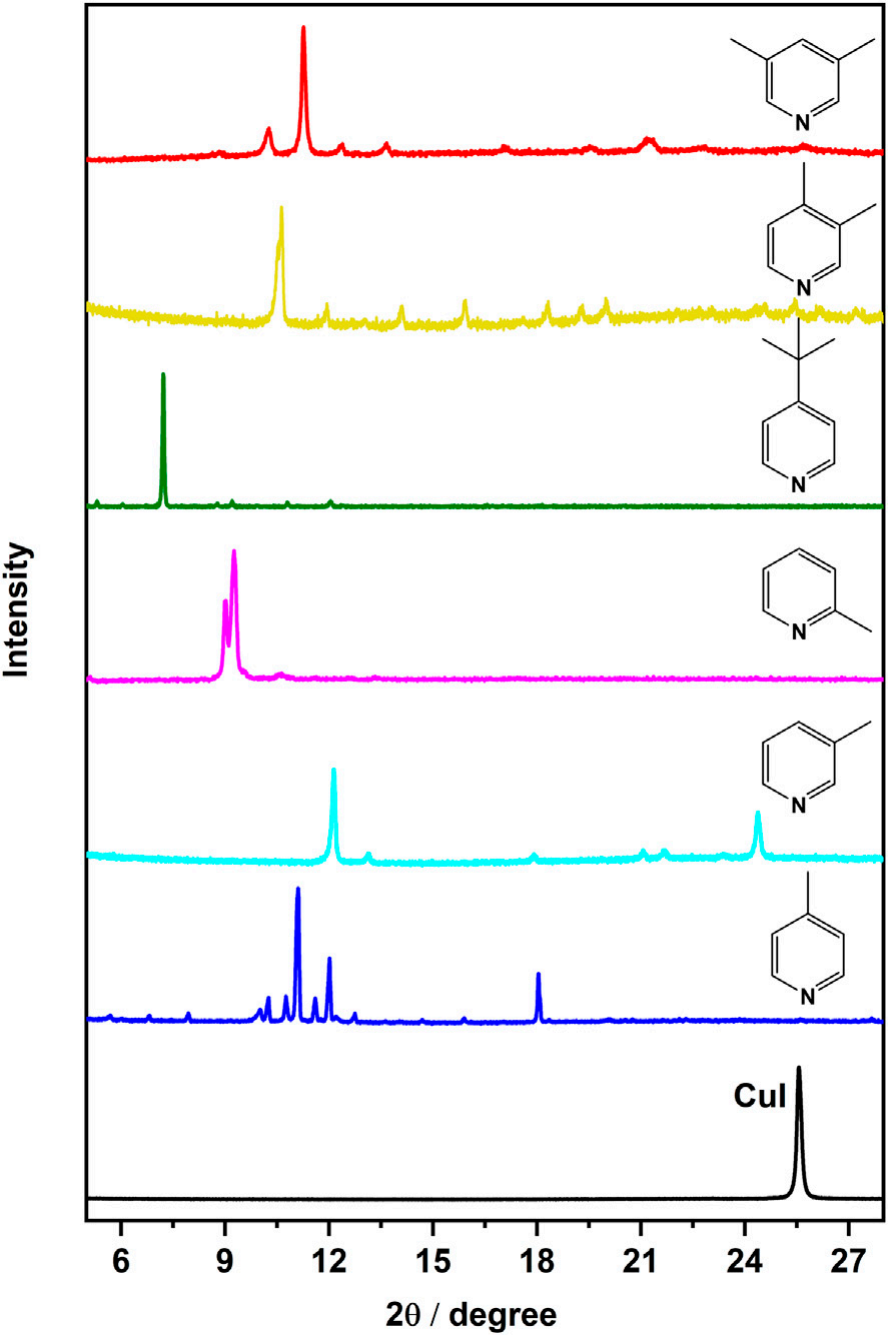
PXRD pattern of CuI film and complexes, [CuI(L)]_n_, on films shown with ligands (L) as prepared by the SILAR method.

In accordance with the experimental section, we utilized the spin coating method to produce thin films of copper(I) iodide on glass substrates. Spin coating is widely recognized for rapidly generating uniform thin films with limited aggregation. Afterward, vapor diffusion as a direct technique for the preparation of copper(I) halide complexes as thin films were utilized. As seen in Figure 2, the X-ray diffractograms confirm the reaction of the ligands with the copper(I) films upon exposure to the ligand by vapor diffusion. No CuI peak is detected after the reactions and there are no broad peaks indicating the formation of amorphous material.

### 3.2 Photoluminescence and coordination reaction

By monitoring the photoluminescence spectra of copper(I) iodide films as a function of exposure time to ligand vapors the reaction coordination processes can be resolved throughout the entire reaction timeline. A detailed analysis of the photoluminescence spectra revealed distinct changes in emission characteristics as the reaction progressed. The emission intensity, peak position, and spectral shape exhibited notable variations over time, indicating the alteration of reaction coordination states on the film surface. Therefore, thin film of CuI was exposed to the vapor of L and the photoluminescence spectra and decay kinetics (lifetime, τ) were measured. The photophysical data and their comparison to the crystalline sample are shown in Table 1.

**TABLE 1:** Photoluminescence maxima and lifetimes of samples in powder and thin films at room temperature.

Compound	Media	λ_max_	τ (µs)	Reference
[CuI(4-Mepy)]_n_	Film	498, 700	5.5	This work
[CuI(4-Mepy)]_4_	Powder	580		Cariati et al. (2000)
[CuI(4-Mepy)]_∞_	Powder	437		Cariati et al. (2000)
[CuI(3-Mepy)]_n_	Film	498	5.5	This work
[CuI(3-Mepy)]_4_	Powder	614	8	Cariati et al. (2000)
[CuI(3-Mepy)]_∞_	Powder	454	0.38, 3.8	Cariati et al. (2000)
[CuI(2-Mepy)]_n_	Film	457, 588		This work
[CuI(2-Mepy)]_4_	Powder	452		Die Strukturchemie der KupferI et al. (1980)
[CuI(2-Mepy)]_∞_	Powder	658		Die Strukturchemie der KupferI et al. (1980)
[CuI(4-^*t*^Bupy)]_n_	Film	500	0.4	This work
[CuI(4-^*t*^Bupy)]_4_	Powder	602		Cariati et al. (2005)
[CuI(4-^*t*^Bupy)]_∞_	Powder	452	0.4, 2.7	Cariati et al. (2005)
[CuI(3,4-diMepy)]_n_	Film	490	0.6	This work
[CuI(3,4-diMepy)]_4_	Powder	667	9.52	Parmeggiani and Sacchetti (2012)
[CuI(3,5-diMepy)]_n_	Film	514	0.5	This work
[CuI(3,5-diMepy)]_∞_	Powder	436		Kitada and Ishida (2014)

**FIGURE 3:**
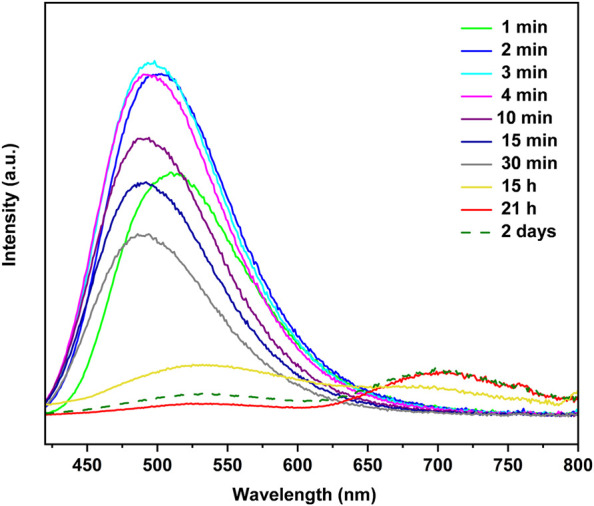
Time dependent photoluminescence for the reaction of a CuI film with 4-Mepy vapors at 25°C in a 10.0 mL vial.

To prepare the thin films of the copper(I) iodide coordination complexes, one CuI film (2 × 0.5 cm^2^) was placed in an empty 2.0 mL vial, which was then placed into a 10.0 mL vial containing a ligand (L). Upon exposure to 4-Mepy vapor, the initially non-emissive CuI film exhibits a remarkable transformation in its emission. Figure 3 illustrates the time-dependent photoluminescence spectra, highlighting the evolution of emission characteristics. Within a few seconds of exposure, a vivid blue emission emerged, resembling the emission exhibited by the [CuI(4-Mepy)]_∞_ polymer as powder. The intensity of the blue emission reached its peak after three minutes, with an emission maximum (λ_em_) at 498 nm.

Subsequently, the blue emission gradually diminished over a period of 15 h, accompanied by the appearance of a low-energy red emission band at 700 nm. This red emission became increasingly dominant after 21 h, suggesting the presence of a tetramer, [CuI(4-Mepy)]_4_, on the film’s surface. Notably, after 2 days, the high-energy band regained dominance, and it remained as the sole emission feature even after 33 days of storage in a sealed vial. The emission disappeared after 2 days of exposure to air, which shows the sensitivity of the stability of the film to environmental pressure.

The reaction of of 3-Mepy with CuI film led to the blue emission at 498 nm, which is characteristic of [CuI(3-Mepy)]_∞_ polymer (Supplementary Figure S1).

**FIGURE 4:**
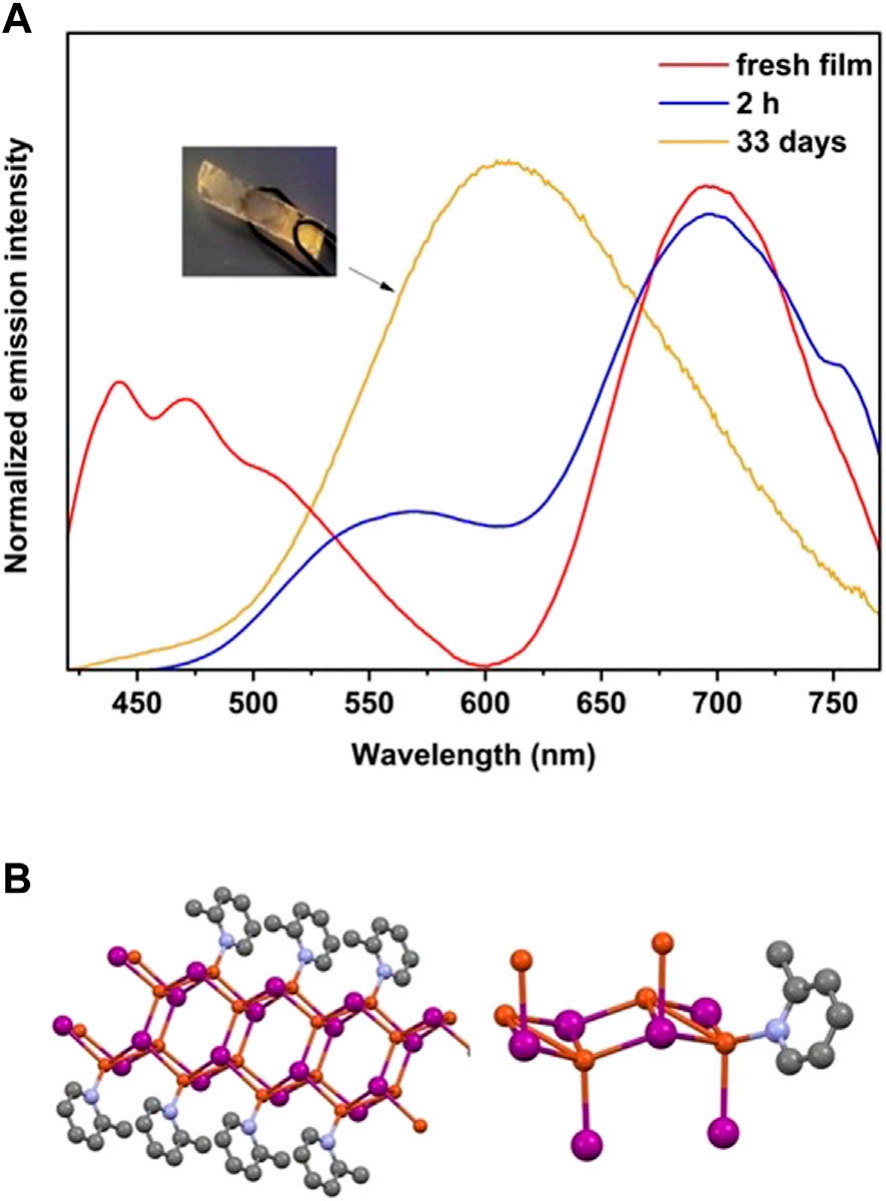
**(A)** Photoluminescence from the reaction of a CuI film with 2-Mepy after 30 min, 2 h and 33 days of exposure in a 10.0 mL vial. The inset shows a picture of the film under illumination after 33 days of exposure to the ligand. **(B)** Crystal structure of [Cu_2_I_2_(2-Mepy)]_n_, which shows red emission at 658 nm.

Based on the information presented in Figure 4, when CuI thin film is exposed to 2-Mepy vapor, a reaction occurs resulting in dual emission, with a dominant high energy peak observed from the initial moments of exposure. The peaks appear to originate from two distinct structures, possibly indicating the formation of two different stoichiometric structures (a tetramer and a polymer). Previous studies suggest that the enduring red emission, which remains stable even after two hours, can be attributed to [Cu_2_I_2_(2-Mepy)]_n_ (Die Strukturchemie der KupferI et al., 1980). The X-ray diffraction patterns (PXRD) of both the film and the powder exhibit a prominent peak at 9.2°. However, when the blue-emitting [CuI(2-Mepy)]∞ film is placed in a sealed vial in contact with vapors at room temperature, a complete transformation to the yellow-emitting cubane [CuI(2-Mepy)]_4_ is achieved within a span of 2 days.

The photoluminescence spectra of the reaction with ^*t*^Bupy vapor exhibits time-dependent spectra that are characterized by a strong and blue emission at 500 nm and decay constant of 0.4 µs, accompanied by a broad and lower energy peak at 700 nm. Interestingly, this behavior contradicts what is observed in the solid state, indicating that the predominant species in the reaction are polymers, with only a small presence of clusters contributing to the excited state known as CC (charge-transfer) (Cariati et al., 2005).

The emission from 3,4-diMepy remains consistent even after being exposed to the ligand for a period of 2 days. The emission is characterized by a vivid blue color and shows high intensity. It appears that the reaction reaches completion within an hour, and even after 2 days of exposure to ambient air, there is no reduction in emission intensity or quenching observed (as depicted in Supplementary Figure S2A through emission spectra and color images). In contrast, when the same reaction is subjected to heating at 70°C, it achieves completion within a mere 2 min (as illustrated in Supplementary Figure S2B). On the other hand, for 3,5-diMepy, the reaction occurs at a significantly faster rate, resulting in the observation of a single green emission peak at 514 nm (as shown in Supplementary Figure S3).

**FIGURE 5:**
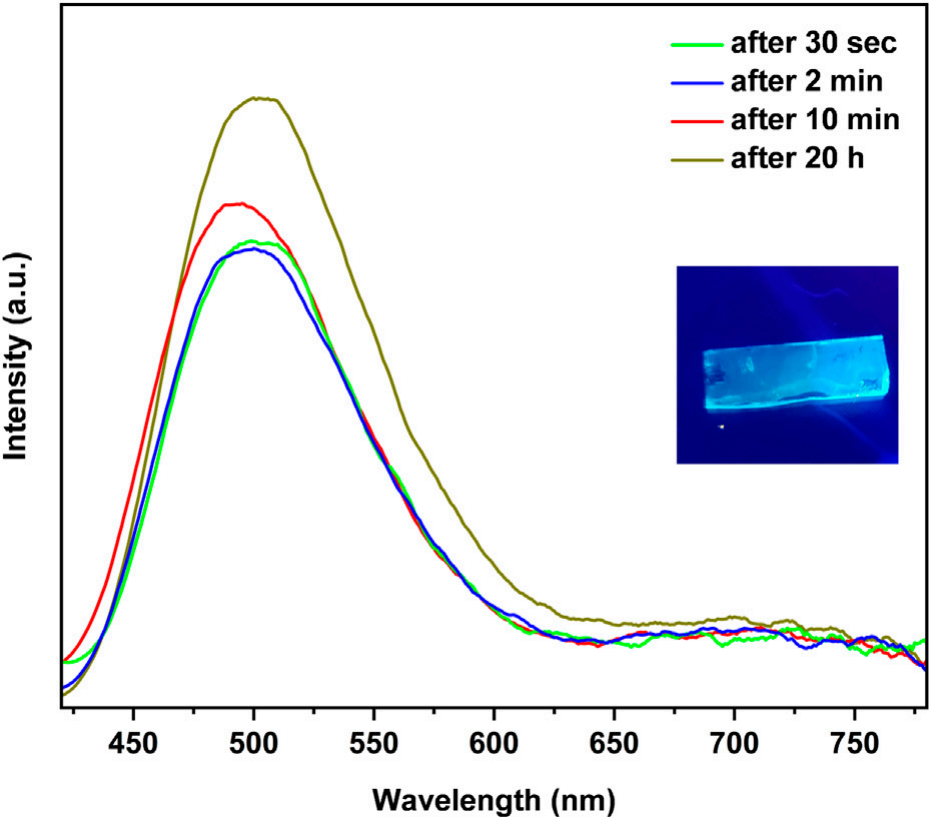
Time dependent photoluminescence of the reaction of CuI film with ^*t*^Bupy vapor at 25°C in a 10.0 mL vial.

Consequently, it appears that for di-substituted pyridine ligands and para-bulky substituted pyridine ligands such as ^*t*^Bupy (Figure 5), the presence of steric hindrance directs the reaction towards a single product. The literature and thermal gravimetry reports on these compounds suggest that degradation occurs as a result of the loss of 1 alkylpyridine ligand or in the case of two-stage degradation of half of the ligands. In such cases, fluorescence was not detectable either at room temperature or at low temperature (De Ahna and Hardt, 1972).

### 3.3 Luminescence responsive to volatile halogenated compounds (VHC)

Having sensors for halogens plays a crucial role in environmental monitoring, health and safety, industrial applications, and emergency response. These sensors help in detecting, quantifying, and tracking halogens, allowing for proactive measures and effective management of halogen-related risks and challenges. To test the viability of copper(I) iodide coordination complexes as sensors for volatile halogenated compounds, we investigated the photoluminescence response from [CuI(3,4-diMepy)]_n_ and [CuI(3,5-diMepy)]_n_ thin films upon exposure to CH_2_Cl_2_ and CH_2_Br_2_.

**FIGURE 6:**
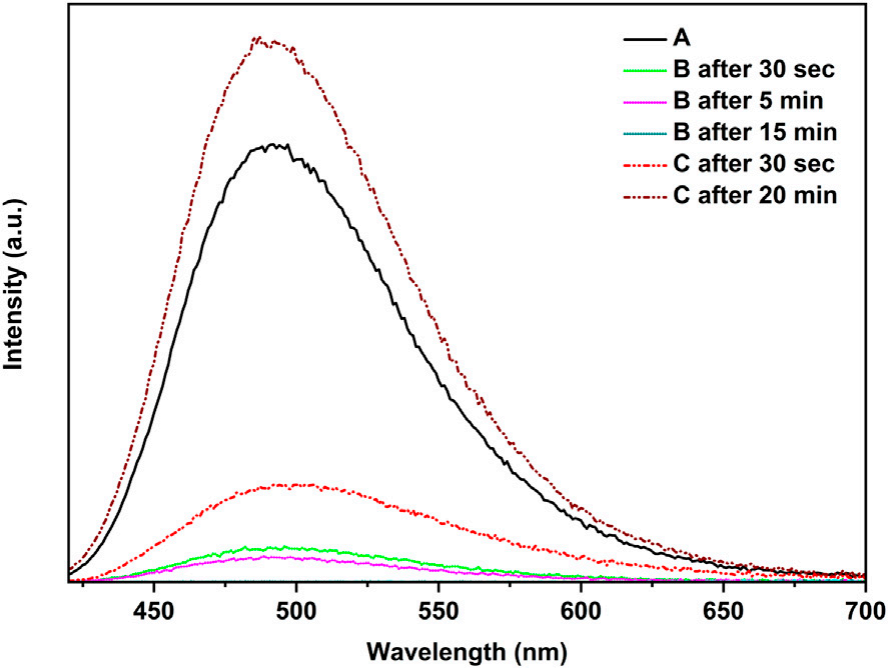
Reaction of [CuI(3,4-diMepy)]_n_, **A**, with CH_2_Cl_2_, **B**, and reverse reaction with 3,4-diMepy, **C**, at 25°C.

Figure 6 demonstrates that when the polymer film [CuI(3,4-diMepy)]_n_ was exposed to a small quantity of dichloromethane vapor, the blue emission was gradually suppressed within a 15-min timeframe. This evidence indicates that the exposure to CH_2_Cl_2_ does not result in the conversion of the polymer to cubane, but rather leads to the emergence of a new non-emissive structure. Indeed, subsequent experiments confirmed this outcome. Conversely, the polymer could be transformed back to CuI(3,4-diMepy)]_n_ by subjecting the sample to 3,4-diMepy vapors for 20 min, resulting in increasing photoluminescence intensity compared to the initial sample.

**FIGURE 7:**
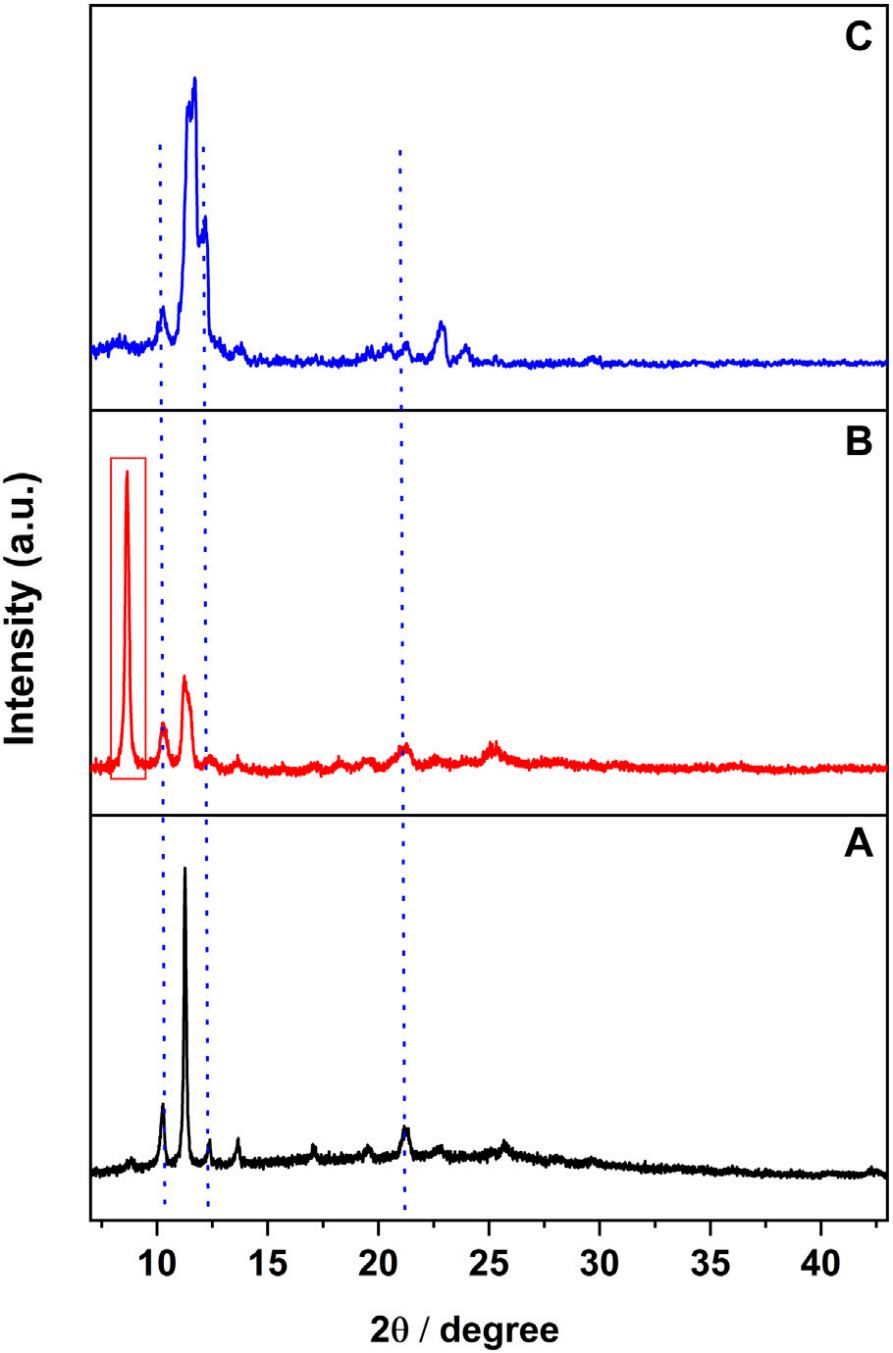
**(A)** PXRD pattern of complex 3,5-diMepy, and **(B)** that after CH_2_Cl_2_ vapor exposure. **(C)** PXRD pattern after exposing sample B to 3,5-diMepy vapor.

Although the PXRD pattern of [CuI(3,4-diMepy)]_n_ samples remained relatively unchanged throughout the process (see Supplementary Figure S4), the PXRD analysis of [CuI(3,5-diMepy)]_n_ (Figure 7A) displayed the appearance of a new peak at 8.7° and broadening of the peak at 11.3° (Figure 7B). Furthermore, after re-exposure to 3,5-diMepy vapor, the peak at 11.3° remained broadened, while the peak at 8.7° vanished (Figure 7C). A similar trend was observed following exposure to CH_2_Br_2_, albeit with a quenching time of approximately 30 min.

**FIGURE 8:**
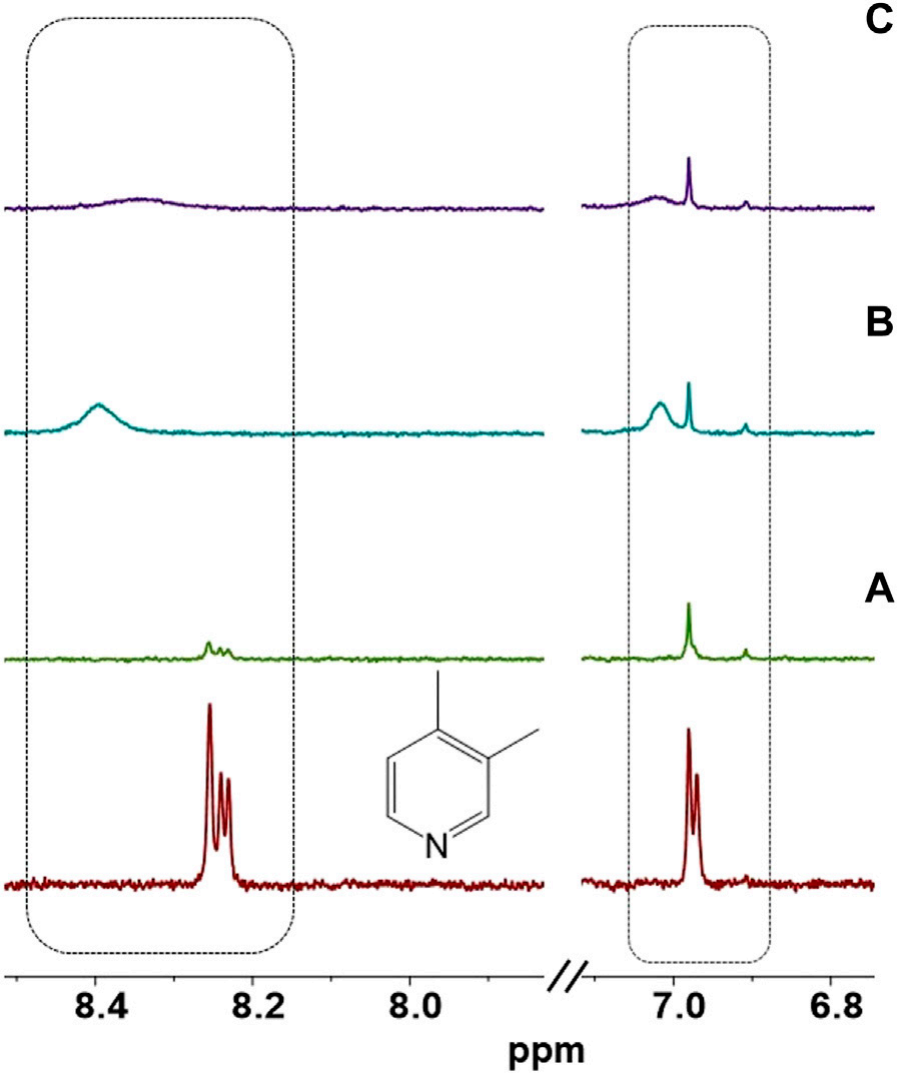
Aromatic region of ^1^H NMR of 3,4-diMepy ligand and **(A)** [CuI(3,5-diMepy)]_n_, and **(B)** after exposure to CH_2_Cl_2_ vapor. **(C)** After re-exposing the film to 3,4-diMepy vapor in CDCl_3_.

To conduct further analysis, the [CuI(3,4-diMepy)]_n_ deposited on the substrate was dissolved in CDCl_3_ and subjected to ^1^H-NMR (Figure 8). The ^1^H-NMR spectrum of the ligand revealed the presence of three distinct hydrogen types in the aromatic region, specifically at chemical shifts of 6.9, 8.22, and 8.23 ppm. It was anticipated that these same peaks would be observed after nitrogen coordination to the copper. Surprisingly, the downfield signals were no longer detectable, and instead, a new broad signal emerged at 8.4 ppm. Additionally, a signal appeared at 7.1 ppm. We postulate that these observations may be indicative of hydrogen bond interactions occurring between the ortho hydrogens of the pyridine ring and the chloride present in CH_2_Cl_2_.

## 4 Conclusion

In conclusion, the fabrication process of thin films of copper iodide alkylpyridine compounds was carried out in a straightforward manner. The characteristics of the resulting structures, their photoluminescence, and stability were found to be influenced by the steric hinderance of the alkylpyridine ligand. It was observed that bulkier ligands led to the formation of a polymer blue emitter structure, which exhibited enhanced stability at ambient temperature.

Additionally, the sensitivity of the films to environmental pressure was demonstrated by the rapid loss of emission within just 2 days of exposure to air. This highlights the crucial importance of considering stability factors when utilizing CuI films in practical applications.

Furthermore, the compounds [CuI(3,4-diMepy)]_n_ and [CuI(3,5-diMepy)]_n_ were specifically investigated as detectors for volatile halogenated compounds. The emission of these compounds was found to be quenched due to structural changes induced by the presence of the volatile compounds. Importantly, the emission could be restored upon re-exposure to the ligand, indicating a reversible behavior.

## Data Availability

The raw data supporting the conclusion of this article will be made available by the authors, without undue reservation.
