# Supplementary material for: Photoluminescent copper(I) iodide alkylpyridine thin films as sensors for volatile halogenated compounds
Source: Front Chem. 2023 Dec 11;11:1330227. doi: 10.3389/fchem.2023.1330227 (PMC10749296; doi:10.3389/fchem.2023.1330227)
Supplement: Supplementary file 1 [file DataSheet1.docx]

Supplementary Material


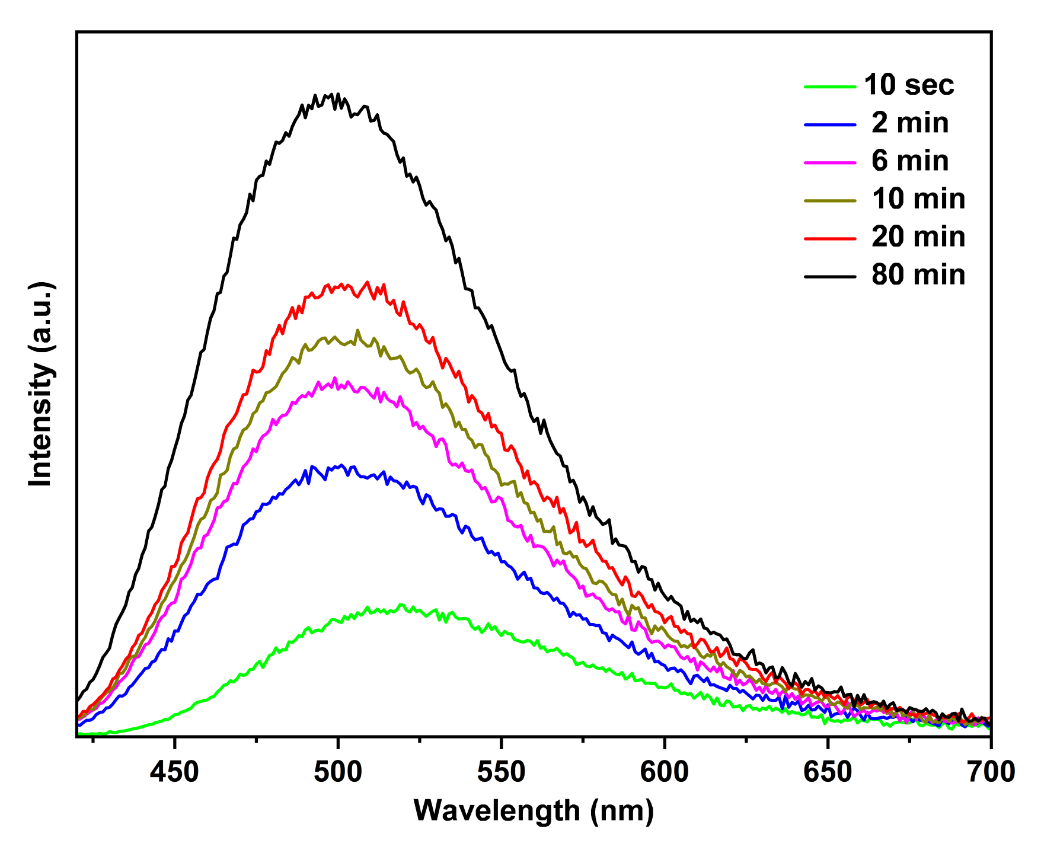


**Figure S1**. Time dependent photoluminescence of the reaction of CuI film with 3-Mepy vapor at 25 °C in a 10.0 mL vial. The intensity increased during the time.


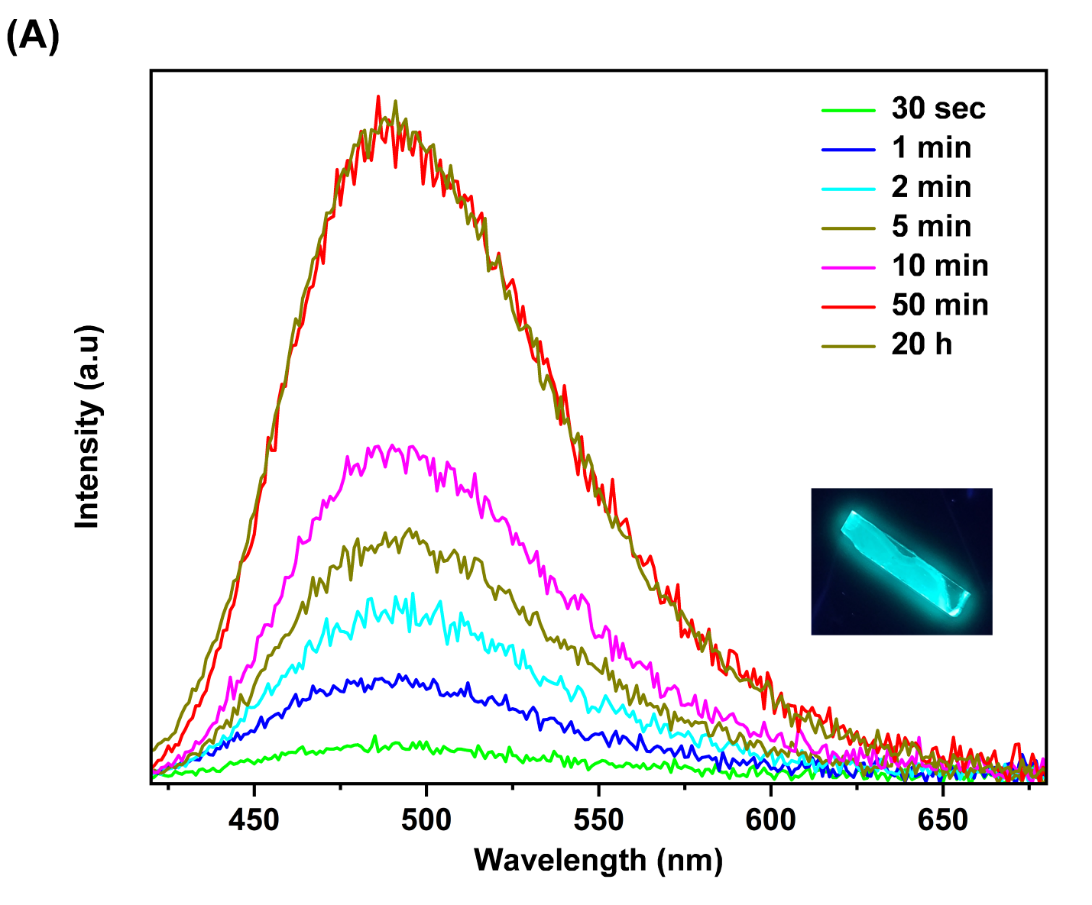


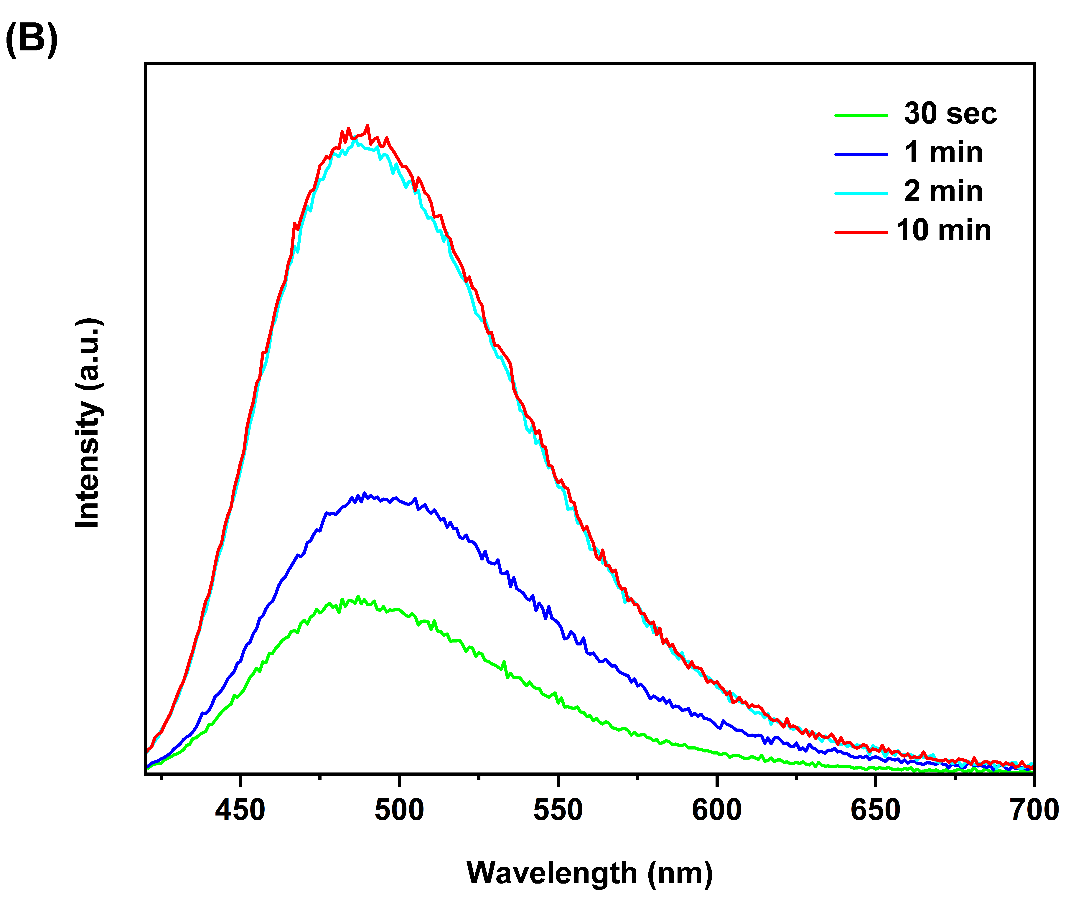


**Figure S2**. (A) Time dependent photoluminescence of the reaction of CuI film with 3,4-diMepy vapor at a) 25 , (B) 70 °C in a 10.0 mL vial. Onset is the emission picture of fresh film.


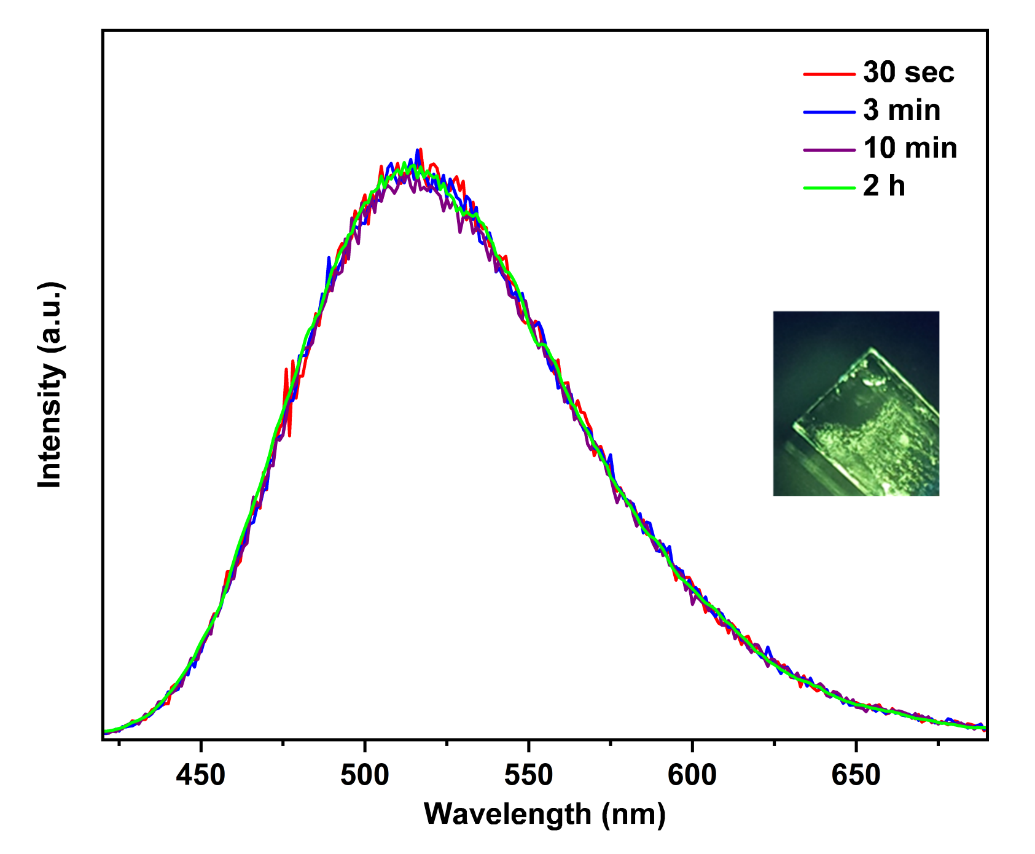


**Figure S3**. Time dependent photoluminescence of the reaction of CuI film with 3,5-diMepy at 25 °C in a 10.0 mL vial. Onset is the emission picture of the fresh film.


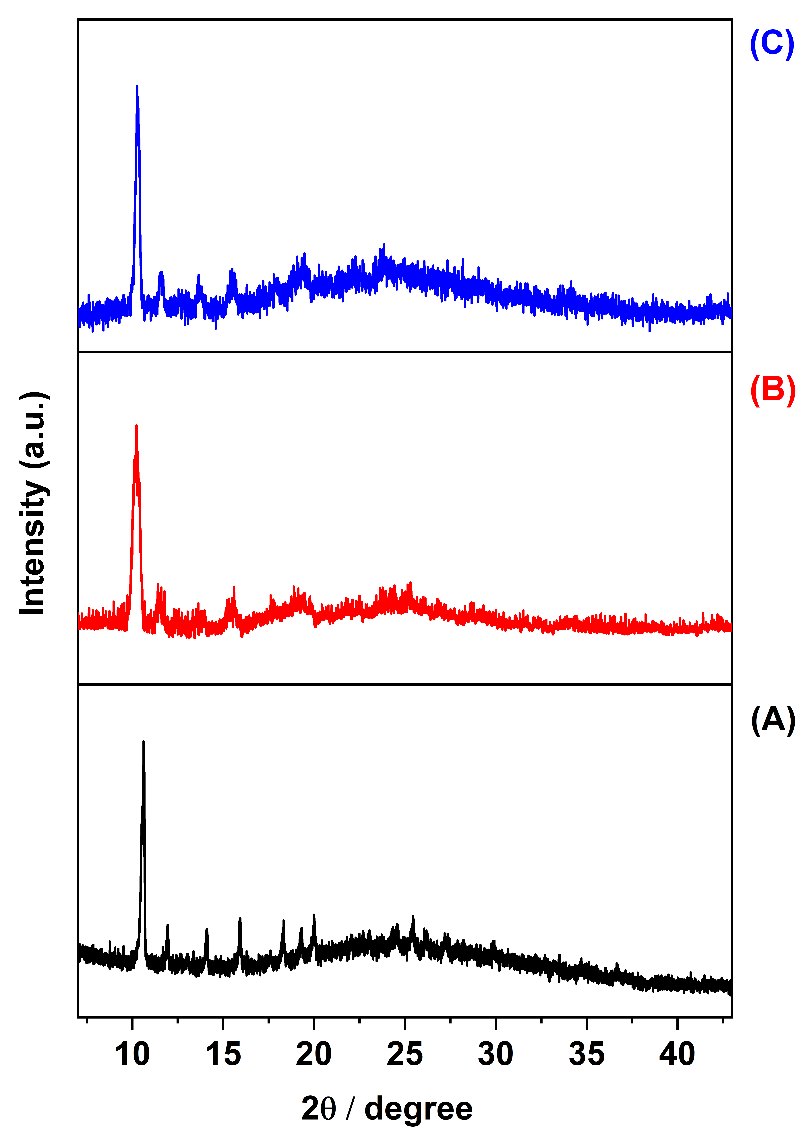


**Figure S4.** (A) PXRD pattern of complex 3,4-diMepy, and (B) that after CH_2_Br_2_ vapor exposure. (C) PXRD pattern after exposing the sample B to 3,4-diMepy vapor.
